# Glutathionyl Hemoglobin and Its Emerging Role as a Clinical Biomarker of Chronic Oxidative Stress

**DOI:** 10.3390/antiox12111976

**Published:** 2023-11-07

**Authors:** Andrea Scirè, Giulia Casari, Brenda Romaldi, Lidia de Bari, Cinzia Antognelli, Tatiana Armeni

**Affiliations:** 1 Department of Life and Environmental Sciences (Di.S.V.A.), Università Politecnica delle Marche, 60131 Ancona, Italy; 2 Department of Odontostomatologic and Specialized Clinical Sciences, Università Politecnica delle Marche, 60131 Ancona, Italy; g.casari@univpm.it (G.C.); b.romaldi@pm.univpm.it (B.R.); t.armeni@staff.univpm.it (T.A.); 3 Institute of Biomembranes, Bioenergetics and Molecular Biotechnologies (IBIOM), National Research Council (CNR), 70126 Bari, Italy; l.debari@ibiom.cnr.it; 4 Department of Medicine and Surgery, Università Degli Studi di Perugia, 06129 Perugia, Italy; cinzia.antognelli@unipg.it

**Keywords:** glutathione, glutathionyl hemoglobin, post-translational modification, S-glutathionylation, glutathionylated hemoglobin, oxidative stress, clinical marker

## Abstract

Hemoglobin is one of the proteins that are more susceptible to S-glutathionylation and the levels of its modified form, glutathionyl hemoglobin (HbSSG), increase in several human pathological conditions. The scope of the present review is to provide knowledge about how hemoglobin is subjected to S-glutathionylation and how this modification affects its functionality. The different diseases that showed increased levels of HbSSG and the methods used for its quantification in clinical investigations will be also outlined. Since there is a growing need for precise and reliable methods for markers of oxidative stress in human blood, this review highlights how HbSSG is emerging more and more as a good indicator of severe oxidative stress but also as a key pathogenic factor in several diseases.

## 1. Introduction

Oxidative stress, caused by an imbalance between oxygen reactive species (ROS) accumulation in cells and the ability of scavenging systems to remove these reactive products, plays a major role in the pathogenesis of a number of diseases. Additionally, the disease severity and progression are linked to the entity which is damaging to various biological structures, namely cellular membranes, lipids, proteins and nucleic acids [1,2]. There are several human diseases where the involvement of oxidative stress has been well documented, including cancer [3,4], diabetes [5], rheumatoid arthritis [6], atherosclerosis [7], cardiovascular disease [8], neurodegenerative disorders such as Parkinson’s, Alzheimer’s and Huntington’s diseases, amyotrophic lateral sclerosis [9,10] and pulmonary, renal, and hepatic diseases [11,12,13]. In the case of neurodegenerative diseases, the initiation or the progression is particularly vulnerable to ROS due to the high metabolic rate, unique lipid composition, and minimal cell turnover of the nervous system. Oxidative stress could also be a causative player in diseases like Alzheimer’s and Parkinson’s disease, amyotrophic lateral sclerosis, and ischemia-reperfusion injuries, considering that copper and iron, able to generate hydroxyl radicals starting from hydrogen peroxide or superoxide, show high levels in specific brain areas [14]. In addition, oxidative stress is well known to be involved in the pathogenesis of certain lifestyle-related disorders, such as hypertension [15], diabetes [16], atherosclerosis [17] and rheumatoid arthritis [18].

Given the strong correlation between oxidative stress and the pathogenesis of several diseases, there is a progressively increasing interest in identifying biomarkers for pathological conditions related to oxidative stress, with the aim of obtaining an accurate evaluation of oxidative stress levels and exploring the efficacy of antioxidant strategies. Among the different systems involved in cell and tissue protection against oxidative damage, glutathione, the main intracellular low-molecular-weight thiol molecule, plays a crucial role. This biological redox agent is able to shield thiol groups of proteins from irreversible oxidation and inactivate ROS, thus reducing and preventing damage caused by free radicals. Glutathione can exist as glutathione (GSH) or glutathione disulfide (GSSG). It can also bind to different proteins through disulfide bond formation, obtaining glutathionylated proteins [19]. Although reversible, this post-translational modification (PTM) has the potential to escalate as a pathological condition, as observed in the S-glutathionylation of actin during prolonged oxidative stress in Friedreich’s ataxia (FRDA). The impact of this continuous modification of actin is to impair its polymerization capacity and its affinity for tropomyosin, compromising the stability of actin filaments and causing a dysregulation of the cytoskeleton [20]. Moreover, in Alzheimer’s disease, S-glutathionylation of glyceraldehyde 3-phosphate dehydrogenase (GAPDH) prevents the correct functioning of this protein involved in the glycolytic pathway [21]. Therefore, considering that specific proteins are involved in each disease, PTMs such as S-glutathionylation could contribute to the manifestation of neurodegenerative diseases as well as other disorders associated with oxidative damage [22,23]. Of the several proteins that may undergo S-glutathionylation, hemoglobin has captured the attention of scientists because of both the high susceptibility of red blood cells to oxidative damage and the pivotal role played by glutathione in protecting erythrocytes from oxidative stress. Indeed, oxidative stress to hemoglobin could induce the formation of hemi-chromes and heme degradation products, thus leading to inflammatory processes and tissue damage [24]. To counteract this harmful scenario, glutathione neutralizes ROS using enzymes, like glutathione peroxidase (GPX) and glutathione reductase (GR), and maintains the redox state of hemoglobin, avoiding the generation of damaging methemoglobin [24]. Starting from this evidence, glutathionyl hemoglobin (HbSSG), in which glutathione binds mainly to the cysteine residue Cys93 of β hemoglobin chain by a disulfide bond, has been largely investigated as a protective response against oxidative damage, and its quantification is vital in clinical research because HbSSG reflects the body’s efforts to counteract the impact of ROS. Indeed, in clinical investigations, HbSSG levels appear to be higher in different oxidative stress-related pathological conditions, including chronic renal failure, diabetes mellitus (DM), hyperlipidemia (HLD), FRDA and iron deficiency anemia (IDA), compared to healthy subjects. Under physiological conditions, about 4% of hemoglobin is glutathionylated, whereas the maximum enhancement of glutathionylation has been detected in DM combined with microangiopathy (69% of HbSSG), suggesting a direct correlation between HbSSG concentration and levels of oxidative stress [19,25,26,27]. Hence, it has been highlighted and confirmed that HbSSG has potential as a biomarker linked to several pathologies related to oxidative stress. [19]. Therefore, starting from an accurate examination of the literature, this review will focus on the potentialities of HbSSG as a clinical serum marker related to oxidative stress conditions, investigating its concentrations in several diseases in which oxidative stress plays a pivotal role and examining various methods used to quantify this modified protein. Indeed, an accurate measurement of HbSSG represents an essential requirement for use as a clinical biomarker, as well as in clinical trials, in order to verify the effectiveness of tested treatments.

## 2. Protein S-Glutathionylation

Glutathione, the main low-molecular-weight cell thiol, is a pivotal antioxidant molecule within mammalian cells, able to contrast oxidizing molecules that alter the cell antioxidant system, such as hydrogen peroxide. Indeed, following the elimination of the oxidizing molecules, GSSG is reconverted into its reduced state (GSH) by GR that uses NADPH as a cofactor [28,29]. The reversible PTM based on the formation of a disulfide bond between the cysteine of glutathione and protein cysteines is called protein S-glutathionylation, and represents one of the S-thiolation forms. This modification is able to communicate changes in the redox state related to GSH and GSSG levels to the cell [28,30]. In particular, S-glutathionylation occurs mostly either by direct oxidation (1) or through the thiol-disulfide exchange process between GSSG and thiol groups of targeted proteins (2) [30,31]. Other mechanisms are possible, because like protein thiols, the cysteine of GSH can be activated through the formation of glutathione sulfenic acid (GS-OH) resulting in protein S-glutathionylation (3) [32,33]. Also, less common mechanisms are described like the thio-sulfinate derivative of GSH (GS(O)SG) or S-nitroso-thiols such as GSNO that could be able to react with protein cysteines or protein thio-sulfinates that could react with GSH [34].

Protein –SH + GSH → Protein-SSG(1)

Protein –SH + GSSG ⇆ Protein-SSG + GSH(2)

Protein –SH + GS-OH → Protein-SSG(3)

In reaction (1) a surface protein sulfhydryl (Protein–SH) becomes activated by ROS through the formation of cysteine sulfenic acid (Protein–S–OH) or cysteine thiyl radical (Protein–S•) and then undergoes S-glutathionylation reacting with GSH. In reaction (2) protein sulfhydryl (Protein –SH) deprotonates to a thiolate anion (Protein–S^−^) in a basic microenvironment, attacking the sulfhydryl of intracellular GSSG, with the subsequent formation of disulfide (Protein –SSG). In reaction (3), besides the formation of glutathione sulfenic acid (GS-OH), the cysteine of GSH can be activated also in the form of glutathione disulfide S-oxide (GS(O)SG) or S-nitroso-thiols such as GSNO [34]. Under physiological conditions, the role of this reversible PTM is to regulate redox signaling cycle and avoid the irreversible modifications of cysteine residues. Instead, following the onset of severe oxidative stress condition, part of GSH is used to eliminate free radicals, thus leading to a reduction in GSH concentration and an increase of GSSG level. The decrease of GSH/GSSG ratio produces a large spectrum of oxidized biological molecules and overwhelms protein S-glutathionylation (mainly through the above-mentioned thiol-disulfide exchange reaction (2)) trying to prevent the redox signaling disruption [35]. S-glutathionylation can occur spontaneously in a oxidized environment or can be catalyzed by enzymatic systems, such as glutathione S-transferase (GST), peroxiredoxins and sometimes glutaredoxins, able to transfer glutathione molecule to targeted proteins [35]. In particular, among the different cytosolic GST, it has been reported that GSTπ, mainly present in the lung and liver but also in the brain and heart, induces both in vitro and in vivo S-glutathionylation [36,37]. Along with GSTπ, the glyoxalase system and glutaredoxin, according to the ratio between GSH and GSSG, enzymatically promote the creation of glutathione adducts [37,38]. Upon returning to normal redox condition, since it is a reversible modification, S-glutathionylation could be reverted by the thiol-disulfide exchange reverse reaction (deglutathionylation), spontaneously or catalyzed by specific enzymes, called protein thiol-disulfide oxidoreductases [39]. In particular, thioredoxin, glutaredoxin and protein disulfide isomerase, belonging to the protein thiol-disulfide oxidoreductase family, represent pivotal enzymes able to catalyze protein disulfide bond reduction. Given that they can occasionally also induce S-glutathionylation under oxidative stress condition, as reported above, these enzymes contribute to the creation of a highly dynamic redox microenvironment [35]. Thioredoxin is involved in pro-proliferative, anti-apoptotic and anti-oxidative mechanisms, and its glutathionylation causes an impairment of its function [40]. Glutaredoxins, also called thioltransferases, possess the activity of thiol-disulfide exchange, thus reducing glutathionyl proteins. Glutaredoxins have also been identified within erythrocytes, in which they promote the formation of hemoglobin starting from HbSSG [41]. Lastly, protein disulfide isomerase contributes to the reduction, formation and isomerization of protein disulfide bonds, crucial for the correct structure and fold of several proteins, within the mammalian endoplasmic reticulum [42]. In most studies, glutathionylation has been found to occur under pathological conditions, including chronic renal failure, DM and atherosclerosis, but it is also present under normal conditions as a set of physiological responses to oxidative stress. In most cells of healthy subjects, glutathione is bound to cytosolic proteins, and approximately half of the glutathione binds to proteins of the endoplasmic reticulum. Therefore, several structural and functional proteins could be targeted by S-glutathionylation, which varies by tissue type, resulting generally in an alteration of their functionalities. Evidence has been reported of reduction of catalytic efficiency of several enzymes, including carbonic anhydrase III in hepatocytes, mitochondrial NADP(+)-dependent isocitrate dehydrogenase and bovine lens aldose reductase, following S-glutathionylation, along with alterations in protein stability and modifications in the capacity of transcription factors to bind targeted DNA sites [19,43]. Moreover, several studies confirm that glutathione plays an important role in cellular dynamics. Indeed, proteins interacting and binding to glutathione (glutathionylated proteins) have been identified in different cells and tissues in multiple physiological and pathological conditions [43].

Overall, S-glutathionylation is a rapid, specific and reversible reaction, which, in addition to its protective function, can modulate cellular functions, such as metabolism, energy sensing, cell migration and growth, based on the redox state in which the cell is found [28,44]. Protein S-glutathionylation occurs both under physiological conditions and pathological states in which defense mechanisms are triggered against oxidative stress.

Quantification of S-glutathionylated proteins can be obtained using different methods characterized by distinct sensitivity and applications. Choice of the most suitable method to be used in different kinds of analysis is strictly related to specificity, sensitivity and the research objectives to be achieved [19]. Research into advancing methods for S-glutathionylation quantification is still ongoing with the aim of advancing redox biology insights and improving patient care.

## 3. S-Glutathionylation of Hemoglobin

**Figure 1:**
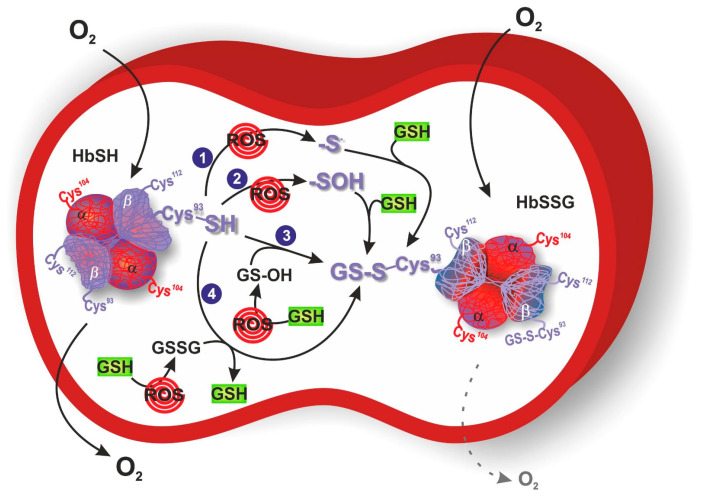
Response of erythrocytes to transient and chronic conditions of oxidative stress via glutathionyl hemoglobin (HbSSG) formation. In mechanism 1 and 2, oxygen reactive species (ROS) catalyze the formation of Hb β-93-cysteine thiyl radical or sulfenic acid, respectively. These intermediates react with reduced glutathione (GSH) to generate HbSSG. In mechanism 3, ROS oxidize GSH to the sulfenic acid form, which reacts with Hb β-93-cysteine to generate HbSSG. In mechanism 4, ROS increase oxidized glutathione (GSSG) concentration that can be attacked by Hb β-93-cysteine in its thiolate anion form. O_2_ dotted arrow represents the reduced oxygen delivery of HbSSG to targeted tissues. Hemoglobin is represented with its glutathionylation sites (αCys104, βCys93 and βCys112), where βCys93 corresponds with the favorite site for S-glutathionylation.

Among the different proteins that could be glutathonylated there is hemoglobin. Human hemoglobin is a tetrameric protein, composed of two α globin chains and two β globin chains (α_2_β_2_), which plays a key role in oxygen transport in red blood cells throughout the body. Indeed, each globin chain contains a heme able to bind one molecule of oxygen. Several intra- and inter-subunit interactions occur between amino acid residues in hemoglobin and are responsible for the dynamic equilibrium between the oxy state (oxygen-bound form, HbFe^2+^-O_2_), and deoxy state (oxygen-free form, HbFe^2+^) of hemoglobin, and its related functions. One cysteine residue is contained in each α globin chain (αCys104), whereas two cysteine residues can be found in each β globin chain (βCys93 and βCys112) (Figure 1). Between these three cysteine residues, αCys104 is totally hidden and βCys93 is far more reachable compared to βCys112. Therefore, βCys93 residue represents the favorite site for the binding of the tripeptide glutathione molecule in order to obtain HbSSG, even if glutathionylation at βCys112 has also been demonstrated [45,46]. This conjugation occurs in response to oxidative stress and serves as a protective mechanism against oxidative stress conditions. Attachment of glutathione to hemoglobin endows it with antioxidant properties, enabling it to scavenge harmful ROS and thwart oxidative damage to cellular components [47]. In the glutathionylation of hemoglobin (HbSH) to glutathionyl hemoglobin (HbSSG), a first possibility is its direct oxidation by ROS to the formation of the β-93-cysteine thiyl radical (Hb-S•) or the β-93-cysteine sulfenic acid (Hb-SOH) that reacts with GSH leading to HbSSG (Figure 1, mechanisms 1 and 2). Another possibility is when hemoglobin acts as a buffer scavenger of oxidized glutathione in its transient form (glutathione sulfenic acid, GS-OH), yielding the mixed disulfide protein HbSSG (Figure 1, mechanism 3) or when, under severe oxidative stress conditions, a higher ratio of GSSG to GSH is present allowing HbSH (in a deprotonated thiolate anion –S^−^ form) to attack the sulfhydryl of GSSG (Figure 1, mechanism 4).

The properties of HbSSG have been studied using different methods, such as nuclear magnetic resonance (NMR) spectroscopy [48], circular dichroism, tryptophan fluorescence, molecular modeling and differential scanning fluorimetry [49]. This oxidative modification induces a shift in the average molecular mass of β globin chain from 15,867 Da of native β globin to 16,172 Da of modified β globin [50]. It has been reported that tertiary structural perturbations of HbSSG, including perturbation of the β-heme pocket, dissociation of the salt bridge between βAsp94 and βHis146 and the hydrogen bond among βAsp99 and αTyr42, were identified by NMR spectroscopy [48]. In addition, quaternary structural modifications of both oxy and deoxy states, such as destabilization of different hydrogen bonds at the αβ interface, have also been detected using hydrogen/deuterium exchange combined with matrix assisted laser desorption ionization mass spectrometry [51]. Instead, recent studies reported that S-glutathionylation of hemoglobin promotes changes in the secondary structure of HbSSG without altering its tertiary and quaternary protein structures, in contrast to the noncovalent complex of hemoglobin with GSH, characterized by an unchanged secondary structure and modified tertiary and quaternary structures [49]. As a result of its conformational changes, HbSSG shows a six-fold increased oxygen affinity compared to normal hemoglobin along with a decreased cooperativity, thus reducing the oxygen delivery to targeted tissues. Enhancement of the oxygen affinity of HbSSG could be related to a modification of the heme position and environment, because binding affinity is strictly associated to heme iron. However, despite the increased conformational flexibility of the modified hemoglobin caused by the elimination of several noncovalent interactions at the interface between α and β globin chains, the overall structure of hemoglobin does not significantly change thanks to the positioning of the glutathione molecule into a groove of tetrameric conformation [50].

## 4. Glutathionyl Hemoglobin and Methemoglobin

HbSSG could also be produced starting from the oxidation of hemoglobin to methemoglobin within intact erythrocytes, thus establishing an indirect interconnection between HbSSG and methemoglobin [52]. In particular, methemoglobin is obtained through the reaction between the Fe^(II)^O_2_-group of oxyhemoglobin and oxidant molecules, like H_2_O_2_, nitrite, nitric oxide and hydroperoxide, which induce the oxidation of hemoglobin iron from the ferrous state (Fe^2+^) to the ferric one (Fe^3+^) [53]. In contrast to normal hemoglobin, methemoglobin is unable to transport oxygen because the transport of oxygen needs a reversible bound between oxygen and ferrous hemoglobin, thus reducing the amount of oxygen delivered to different tissues and enhancing the potential risk of tissue hypoxia [54]. Moreover, production of methemoglobin within healthy erythrocytes promotes erythrocyte disfunctions, like peroxidative changes in their membranes [52]. Under physiological conditions, methemoglobin is produced at a 3%/day rate since, although the oxygenated hemoglobin is a stable molecule, it slowly undergoes an auto-oxidization process [54]. However, several protective mechanisms, like the cytochrome b5-methemoglobin reductase pathway, maintain its levels below 1% by reducing the ferric ion (Fe^3+^) to the ferrous ion (Fe^2+^). Indeed, cytochrome b5 reductase is able to convert methemoglobin to hemoglobin through the nicotinamide adenine dinucleotide, thus eliminating 95–99% of methemoglobin [55]. Furthermore, NADPH-methemoglobin reductase, involved in another enzymatic pathway, is able to neutralize methemoglobin, thus removing the remaining 5% of produced methemoglobin. This second pathway could also be pharmacologically activated by other cofactors, including methylene blue [56]. The pathological condition related to high levels of methemoglobin in the blood, induced by drugs or toxins, is called methemoglobinemia and could lead to a fatal tissue hypoxia [53].

Investigating the connection between methemoglobin and HbSSG, it has been reported that incubation of erythrocytes with the cell membrane permeable hydroperoxide tert-Butyl-hydroperoxide (tBHP) linearly enhances the formation of methemoglobin until 10 min of incubation (4), which in turn reacts with GSSG to form HbSSG (5). Instead, the sulfhydryl reductant dithiothreitol (DTT) avoids the production of methemoglobin by tBHP, protecting the erythrocytes from peroxidative changes (6) [52].

hemoglobin + tBHP → methemoglobin(4)

methemoglobin + GSSG → HbSSG(5)

(6) $$\mathrm{hemoglobin}+\mathrm{tBHP}+\mathrm{DTT}\;\overset{\mathrm{no}\;\mathrm{reaction}}{\to }\;\mathrm{methemoglobin}\;\overset{\mathrm{no}\;\mathrm{reaction}}{\to }\;\mathrm{HbSSG}$$

Therefore, different kinds of hemoglobin glutathionylation can occur within intact erythrocytes, including the formation of a mixed disulfide bond among GSH and normal hemoglobin, but also between cysteine residues of methemoglobin and GSSG [57].

## 5. Methods for the Detection of Glutathionyl Hemoglobin

In order to better highlight both the physiological and pathological role of glutathionylated proteins, including hemoglobin, several studies have been focused on the methodological systems used to quantify them.

Usually, quantification of S-glutathionylated proteins is achieved by measuring the amount of GSH released after reduction of the disulfide bond. This analysis can be carried out by spectrophotometric assays, that generally consists in the quantification of GSH after reduction of the protein mixed disulfide [58], but most of the procedures applied for the detection of S-glutathionylated proteins rely on high performance liquid chromatography (HPLC) separation to increase the sensitivity of the assay and to decrease the detection limit. The chromatographic run is generally coupled to spectrophotometric or fluorometric detection of GSH, which has been tagged with the revealing of molecules [59,60,61]. The presence of S-glutathionylated proteins can also be revealed by conventional mass spectrometry (MS) procedures. In this case, the mass difference due to GSH binding can be verified by direct electrospray ionization (ESI) measurements in intact proteins [62]. As an alternative to HPLC–ESI–MS analyses, a novel technique based on linear mode matrix-assisted laser desorption/ionization time-of-flight mass spectrometry (MALDI-TOF MS) has been developed for the quantitative determination of HbSSG in blood samples [63,64]. There are also qualitative methods to detect S-glutathionylation, such as the use of specific antibodies [65], or specific chemical labeling like the use of biotin [66].

Analysis of glutathionylated proteins is challenging to perform because their concentrations could be overestimated due to GSH artefactual oxidation in the pre-analytical step, thus leading to an increase in both GSSG and glutathionylated protein levels. With the aim of preventing this experimental trouble, it has been recently developed a specific HPLC-based method by [67]. Briefly, after collection, samples have been pre-treated with an alkylating agent, then protein deglutathionylation has been induced by DTT, and the released GSH, previously marked with a fluorescent probe, has been successfully quantified by HPLC. Thiol groups of proteins are thus protected from their artificial oxidation. The main disadvantage of this method is the inability to discriminate between the various glutathionylated proteins, thus not allowing identification of each specific protein subjected to S-glutathionylation, whereas the main advantage is the possibility of accurately estimating the total quantity of glutathionylated proteins in the analyzed sample. The protocol applied by [67] can be considered for all biological samples. However, as far as blood is concerned, it appears to be particularly subject to the autoxidation of GSH, due to the presence of oxygen and iron within the heme group. Therefore, by applying this protocol in the blood, the data loses some significance, unlike the data obtained from other biological samples. Hence, investigation and development of specific techniques have become necessary for a more accurate detection and quantification of glutathionylated proteins within the blood [67]. Starting from this evidence and focusing specifically on HbSSG, it has been reported that its detection is not possible within healthy erythrocytes using traditional analytic techniques, like electrophoresis, because of the low concentrations of GSSG in normal cells. Instead, highly specific and sensitive techniques, like HPLC-ESI-MS, are able to detect HbSSG also in normal erythrocytes [68]. In particular, liquid chromatography combined with mass spectrometry separates HbSSG from other hemoglobin variants and then subjects it to mass spectrometry for precise quantification. However, HPLC-ESI-MS is not easily accessible because of the high instrumentation costs, shows low tolerance for impurities in sample preparation and is challenging to use for standard measurements [69]. Therefore, alternative methods, including cation-exchange HPLC with UV detection and MALDI-TOF-MS, have been developed to analyze HbSSG levels, overcoming the limitations of currently used techniques. The first one is a reproducible and simple procedure, based on the separation of HbSSG thanks to its chemical properties and then its quantification using UV or fluorescent detection. It is able to detect concentrations of this post-translational modified hemoglobin in blood, avoiding arduous sample preparation, in order to investigate oxidative stress conditions and evaluate the potential effectiveness of antioxidant strategies. Robustness and reproducibility of this method make it suitable for clinical investigations [69]. The second one is mainly used to quantify the levels of hemoglobin species, like glycated hemoglobin and HbSSG, in blood samples, thus obtaining a quantitative analysis of a potential biomarker of oxidative stress in several pathologies. Moreover, it is characterized by high sensitivity and resolution, and allows for low-cost analysis, enabling the evaluation of a large number of samples in a short time [63,64]. Lastly, hydrogen/deuterium exchange based MS represents a potent technique able to explore the solution-phase conformational dynamics, and hence correlate functional aberrations with structural modifications of the HbSSG [51].

In contrast to healthy erythrocytes, oxidative stress that characterizes several pathological conditions promotes an increase in GSSG levels, giving rise to an enhanced concentration of HbSSG in patient blood, which could be more easily detected and quantified using the above-mentioned techniques [70]. Hence, HbSSG is frequently employed to gauge the extent of oxidative stress in several pathological states.

## 6. Glutathionyl Hemoglobin and Diseases

Elevated levels of HbSSG have been registered in several diseases associated with oxidative stress, including chronic renal failure, DM, HLD, FRDA and IDA, probably due to the severe oxidative stress within erythrocytes of patients affected by these conditions (Table 1). Among the several disorders related to oxidative damage, uremia is a pathological condition that occurs in advanced states of chronic renal failure. Patients affected by this disease may undergo hemodialysis (HD) or continuous ambulatory peritoneal dialysis (CAPD) as therapeutic strategy [71]. Uremic patients show a higher ratio of GSSG to GSH, in which GSSG represents a necessary source for the transformation of hemoglobin into HbSSG, whereas GSH within red blood cells prevents hemoglobin from being oxidized into methemoglobin. Several studies have taken into consideration patients who have undergone HD, following which there is an increase in HbSSG compared to normal subjects, probably due to the greater oxidative stress. Indeed, an excess of oxidative stress occurs after HD treatment because of both the poor or defective antioxidant defense and the activation of polymorphonuclear neutrophils through a contact system between blood and dialysis membranes, thus leading to a low concentration of GSH, an increase in GSSG and a lower activity of glutathione-dependent enzymes (GST, GR, etc.) in the red blood cells [27,72]. Other than HD, uremic patients could be treated with CAPD. Therefore, HbSSG levels have also been analyzed in this pathological condition after CAPD treatment by HPLC-ESI-MS. Concentrations of HbSSG significantly increase in CAPD patients, as well as HD patients, compared with healthy subjects. However, no statistically significant differences have been identified in HbSSG levels between HD and CAPD-treated patients [27]. To conclude, the redox balance of intracellular GSH is an important factor as it determines the good functioning of proteins with respect to oxidative stress. In particular, the quantification of HbSSG could be a useful clinical marker of oxidative stress in chronic replacement therapies, such as HD or CAPD [71]. In addition to uremia, diabetes can be further compromised by oxidative stress, a pathogenic factor involved in diabetic complications, thus causing alterations of lipids, proteins and nucleic acids localized on the cell membrane. DM often causes an increase in oxidative stress with the rise of GSSG within red blood cells. In turn, GSSG forms a disulfide bond with the cysteine β93 of hemoglobin, thus leading to the formation of HbSSG. It has been demonstrated that HbSSG levels are higher in patients with DM compared to healthy subjects, and the administration of vitamin E for eight weeks could lead to the reduction of HbSSG concentrations. Therefore, oxidative stress in red blood cells also determines the increase in HbSSG in patients suffering from DM [62,73]. Furthermore, a minor glutathionyl hemoglobin subfraction, called HbSSG A_1d3_ has also been investigated, comparing diabetic patients and healthy subjects. HbSSG A_1d3_ formation occurs when the amino terminal valine residue of the hemoglobin β chain is covalently derivatized with an Amadori product via the Maillard reaction. The study highlights the characterization of HbSSG A_1d3_ as a subfraction of HbSSG, showing higher levels of this modified hemoglobin in DM patients compared to non-diabetic individuals. In conclusion, HbSSG A_1d3_ could also be employed as a useful marker of oxidative stress in patients suffering from DM [74]. Oxidative stress represents a pathogenic factor not only for diabetic conditions but also for HLD, in which ROS are largely produced and the antioxidant defense system is altered, resulting in a reduced activity of superoxide dismutase (SOD) and weak concentrations of GSH within erythrocytes [75]. Therefore, levels of HbSSG have been investigated in erythrocytes of HLD patients by HPLC-ESI-MS. Increased concentrations of HbSSG have been reported in patients affected by this disease compared with healthy controls, probably related to the enhanced oxidative stress conditions that characterize HLD [62]. Moreover, the oxygen affinity of HbSSG, prepared in vitro through the incubation of hemoglobin with GSH, is higher than both control hemoglobin and hemoglobin incubated without GSH because of the perturbation of its β chain tertiary structure, whereas its cooperativity (heme–heme interaction) is reduced [62]. This experimental evidence suggests that HbSSG in HLD could result in a decreased tissue oxygen supply and thus tissue hypoxia. Oxidative stress also plays a crucial role in various neurological diseases, including FRDA, a neurodegenerative disorder related to the deficiency of a mitochondrial protein involved in iron metabolism regulation, called frataxin [76]. Considering that a perturbation of glutathione homeostasis could result to or from the oxidative stress condition, total, free and protein-bound glutathione levels have been measured in the blood of patients affected by FRDA by [25], thus investigating glutathione role in the pathophysiology of this neurodegenerative condition. A statistically significant increase of HbSSG has been detected in FRDA patient blood compared to healthy subjects by HPLC-ESI-MS, together with a decreased free glutathione concentration (both GSH and GSSG) and comparable total glutathione levels. Hence, reduction of free glutathione levels appears to be related to the corresponding increase of its protein-bound form [25]. Starting from this evidence, further studies are needed to confirm the emerging role of HbSSG as a clinical biomarker of chronic oxidative stress in neurodegeneration. In addition to FRDA, neurodegeneration in several disorders, including Down syndrome (DS), may be promoted by an impairment of antioxidant systems. DS, characterized by a trisomy of the 21st chromosome, represents the leading cause of human mental retardation. Children affected by this disease show enhanced lipid peroxidation, DNA oxidative damage, and increased levels of CuZn SOD [77]. Considering the involvement of glutathione and related enzymes in neuronal cell death mechanisms, HbSSG has been investigated in the blood of DS patients. Levels of both total glutathione and free glutathione, together with concentrations of HbSSG, decrease in blood of children affected by DS, compared with controls [78]. Indeed, neurodegeneration, oxidative stress and glutathione appear to be strictly associated, as confirmed by these results. However, it is still unknown whether the reduction of all glutathione parameters could be attributed to a decreased glutathione synthesis or an enhanced consumption. Furthermore, SOD/GPX activity ratio increases, whereas activities of GST and GR do not show any statistically significant variations than those in healthy subjects [78]. Hence, association of the disequilibrium between SOD and GPX activities and a deficiency in the glutathione system may be responsible for different pathological features of DS. Nevertheless, additional studies are needed to better highlight the role of HbSSG in DS children, given that, in contrast to the majority of other disorders, DS induces a reduction of HbSSG levels. Levels of HbSSG have also been investigated by MS in patients affected by IDA, in which a marked oxidative stress condition, induced by the enhanced oxidants and reduced antioxidants, was registered [79]. IDA patients showed a significant increase of HbSSG compared to healthy controls. Moreover, a positive correlation of HbSSG concentrations has been observed with serum transferrin receptor, and a negative one with serum ferritin, suggesting that its levels represent an indirect biomarker of the amount of the total body iron in IDA [80]. Another disease whose pathophysiology is related to oxidative stress is the major depressive disorder (MDD), a common psychiatric condition characterized by the reduction of vitamin C and SOD levels, along with the increase of reactive pro-oxidants, compared to healthy subjects [81]. Here, levels of HbSSG were quantified by HPLC-ESI-MS in patients affected by MDD and treated or not with selective serotonin reuptake inhibitors for six weeks. Elevated levels of HbSSG were registered compared to controls, whereas treatment with antidepressant medication does not appear to influence its concentration, probably because of the short treatment duration and moderate sample size [82]. Further investigation with a larger sample size and longer antidepressant treatment duration will clarify the role of HbSSG in the MDD condition.

Finally, together with the disorders mentioned above, protein-bound glutathione was investigated in blood samples of cigarette smokers to further confirm the potential of HbSSG as oxidative stress biomarker. Indeed, exposure to cigarette smoke (CS) is the leading cause of chronic oxidative stress, and most of the glutathione bound to proteins in blood is located within red cells in the form of HbSSG [83]. In particular, concentrations of HbSSG are higher in smokers compared to nonsmokers, and directly related to the number of cigarettes smoked per day, blood cotinine and blood thiocyanate [84]. These results are also confirmed by [85], using multistage mass spectrometry able to quantify the amount of glutathionylation at the peptide level in one drop of blood and thus identifying all three cysteine residues that could be glutathionylated [85].

**Table 1:** Summarization of clinical studies that measured the levels of glutathionyl hemoglobin (HbSSG) in different diseases compared to healthy subjects.

Disease/Treatment	Values	Reference
HD	HbSSG β (%)	
Normal n = 20	3.7 ± 0.3	
HD n = 10	18.6 ± 0.9 ^a^*	Naito et al. (1999) [72]
HD n = 10	20.8 ± 0.9 ^b^*	
Normal n = 20	3.0 ± 1.6	
HD n = 30	8.0 ± 3.6 *	
HD n = 12	8.7 ± 3.2 ^a^*	Takayama et al. (2001) [27]
HD n = 12	8.7 ± 2.8 ^b^*	
CAPD	HbSSG β (%)	
Normal n = 20	3.0 ± 1.6	Takayama et al. (2001) [27]
CAPD n = 10	5.9 ± 2.7 *	
DM	HbSSG β (%)	
Normal n = 20	3.7 ± 0.3	
DM n = 10	10.2 ± 0.8 ^c^*	Naito et al. (2000) [73]
DM n = 10	4.1 ± 0.4 ^d^#	
Normal n = 20	3.7 ± 0.3	
DM n = 37	7.9 ± 0.5 *	Niwa et al. (2000) [62]
	HbSSG A_1d3_ (%)	
Normal n = 9	1.2 ± 0.1	Al-Abed et al. (2001) [74]
DM n = 20	2.3 ± 0.3 *	
HLD	HbSSG β (%)	
Normal n = 20	3.7 ± 0.3	Niwa et al. (2000) [62]
HLD n = 17	8.1 ± 0.8 *	
FRDA	HbSSG β (%)	
Normal n = 20	8.0 ± 1.8	Piemonte et al. (2001) [25]
FRDA n = 14	15.0 ± 1.5 *	
DS	HbSSG β (%)	
Normal n = 64	2.65 ± 1.1	Pastore et al. (2003) [78]
DS n = 46	1.47 ± 0.6 *	
IDA	HbSSG β (%)	
Normal n = 15	7.7 ± 3.7	Shet et al. (2012) [80]
IDA n = 23	16.9 ± 9.6 *	
MDD	HbSSG β (%)	
Normal n = 17	5.73	Mathew et al. (2019) [82]
MDD n = 26	8.34 *	
MDD n = 11	8.07 ^e^	
MDD n = 11	7.68 ^f^	Mathew et al. (2019) [82]
CS	HbSSG β (%) ^g^	
Nonsmokers n = 354	5.6	Muscat et al. (2004) [84]
Smokers n = 97	8.1 *	
	HbSSG α (%) ^h^	
Nonsmokers n = 20	2.24 ± 0.91	Chen et al. (2014) [85]
Smokers n = 20	3.61 ± 1.41 *	
	HbSSG β (%) ^i^	
Nonsmokers n = 20	3.79 ± 1.42	Chen et al. (2014) [85]
Smokers n = 20	6.69 ± 2.33 *	
	HbSSG β (%) ^l^	
Nonsmokers n = 20	0.54 ± 0.68	Chen et al. (2014) [85]
Smokers n = 20	0.56 ± 0.39	

HD: hemodialysis, ^a^ before hemodialysis, ^b^ after hemodialysis; CAPD: continuous ambulatory peritoneal dialysis; DM: diabetes mellitus, ^c^ before vitamin E, ^d^ after vitamin E; HLD: hyperlipidemia; FRDA: Friedreich’s ataxia; DS: Down syndrome; IDA: iron deficiency anemia; MDD: major depressive disorder, ^e^ pre-antidepressant treatment, ^f^ post-antidepressant treatment; CS: cigarette smoker, ^g^ estimated value, ^h^ glutathionyl hemoglobin at α–Cys-104, ^i^ glutathionyl hemoglobin at βCys-93, ^l^ glutathionyl hemoglobin at β–Cys-112; * statistically significant differences (*p*-value ≤ 0.05) between pathological conditions and healthy controls; # statistically significant differences (*p*-value ≤ 0.05) between pre- and post-treatment conditions.

In summary, in contrast to the ratio between GSSG and GSH, blood levels of glutathione bound to proteins are stable for a long time, even if glutathionylation represents a reversible reaction, demonstrating that protein glutathionylation, and in particular HbSSG, is a suitable low-invasive marker for chronic oxidative stress in the whole body.

## 7. Conclusions

Starting from the evidence reported within this review, hemoglobin represents one of the most susceptible proteins to undergo the reversible PTM of S-glutathionylation following oxidative stress conditions. Levels of HbSSG show a statistically significant increase in several pathological conditions related to oxidative stress, confirming its emerging role as a clinical biomarker of severe oxidative stress but also as a pivotal pathogenic factor in different diseases. Indeed, in contrast to GSSG levels, which also increase during oxidative perturbations, HbSSG is less susceptible to enzymatic reduction, and therefore more suitable to be used as oxidative stress indicator. Moreover, S-glutathionylation of hemoglobin induces conformational modifications in its structure, resulting in a regulation of hemoglobin functions, such as an increased oxygen affinity and reduced cooperativity compared to non-modified hemoglobin, thus suggesting a role in the pathophysiology of oxidative stress-associated disorders. However, additional studies are recommended to better highlight the role of HbSSG in correlation with oxidative damage and to develop increasingly accurate, as well as reliable, techniques for its quantification in clinical investigations.

## Data Availability

Not applicable.
